# Serum Nostrin—A risk factor of death, kidney replacement therapy and acute kidney disease in acute kidney injury

**DOI:** 10.1371/journal.pone.0299131

**Published:** 2024-04-11

**Authors:** Stefan Erfurt, Martin Lauxmann, Katharina Asmus, Stefanie Oess, Daniel Patschan, Meike Hoffmeister

**Affiliations:** 1 Brandenburg Medical School Theodor Fontane, Institute of Biochemistry, Brandenburg an der Havel, Germany; 2 Department of Internal Medicine I—Cardiology, Nephrology and Internal Intensive Medicine, Brandenburg University Hospital, Brandenburg Medical School Theodor Fontane, Brandenburg an der Havel, Germany; 3 Faculty of Health Sciences (FGW), Joint Faculty of the University of Potsdam, The Brandenburg Medical School Theodor Fontane and the Brandenburg Technical University, Cottbus-Senftenberg, Germany; CSIR-Indian Institute of Chemical Biology, INDIA

## Abstract

**Background:**

The prediction of Acute Kidney Injury (AKI)-related outcomes remains challenging. Persistent kidney excretory dysfunction for longer than 7 days has been defined as Acute Kidney Disease (AKD). In this study, we prospectively quantified serum Nostrin, an essential regulator of endothelial NO metabolism, in hospitalized patients with AKI.

**Design, setting, participants, & measurements:**

In-hospital subjects with AKI of various etiology were identified through the in-hospital AKI alert system of the Brandenburg University Hospital. Serum Nostrin, and serum NGAL and KIM-1 were measured within a maximum of 48 hours from the timepoint of initial diagnosis of AKI. The following endpoints were defined: in-hospital death, need of kidney replacement therapy (KRT), recovery of kidney function (ROKF) until discharge.

**Results:**

AKI patients had significantly higher serum Nostrin levels compared to Controls. The level of serum Nostrin increased significantly with the severity of AKI. Within the group of AKI patients (n = 150) the in-hospital mortality was 16.7%, KRT was performed in 39.3%, no ROKF occurred in 28%. Patients who required KRT had significantly higher levels of serum Nostrin compared to patients who did not require KRT. Significantly higher levels of serum Nostrin were also detected in AKI patients without ROKF compared to patients with ROKF. In addition, low serum Nostrin levels at the timepoint of AKI diagnosis were predictive of in-hospital survival. For comparison, the serum concentrations of NGAL and KIM-1 were determined in parallel to the Nostrin concentrations and the results confirm the prognostic properties of serum Nostrin in AKI.

**Conclusions:**

The current study suggests serum Nostrin as novel biomarker of AKI-associated mortality, KRT and Acute Kidney Disease.

## Introduction

Acute kidney injury (AKI) evolves in a substantial number of in-hospital patients (up to 30%), the incidence may even surpass 60% in intensive care units [1, 2].

In the latter, mortality can exceed 50%, particularly in septic individuals [1]. In addition to decreasing the life expectancy in the short-term, AKI increases the risk of both, chronic kidney disease (CKD) and mortality over the years to come [2, 3]. The time cutoff between AKI and CKD has been defined with 3 months [4]. In AKI, kidney function may either recover completely or incompletely or not at all. The criteria used for the definition of recovery of kidney function (ROKF) are heterogenous [5]. However, the term Acute Kidney Disease (AKD) was already introduced in the 2012 published ´KDIGO clinical practice guidelines for acute kidney injury´ [6]. It reflects persistent excretory dysfunction from day 8 after AKI has been established.

The diagnosis of AKI is still based on the 2012 published KDIGO guidelines [6] and at least one of three criteria listed must be fulfilled. Criteria 1 and 2 are related to dynamic changes in serum creatinine, the third criterion describes a reduction in urine output. Due to the substantial limitations of serum creatinine in terms of early AKI diagnosis and the lack of specificity and prognostic value of serum creatinine, new or alternative AKI biomarkers have been studied extensively over decades. In 2020, Ostermann et al. [7] published the ´Recommendations on Acute Kidney Injury Biomarkers From the Acute Disease Quality Initiative Consensus Conference´. The article weighs the clinical usability of numerous AKI biomarkers of structural damage / function / stress in relation to five variables: AKI risk assessment, prediction of AKI, diagnosis of AKI, the severity of AKI and renal restitution or recovery of kidney function (ROKF). Two important outcome categories were however omitted, in-hospital mortality and the need of kidney replacement therapy (KRT).

The F-BAR protein Nostrin (NO synthase traffic inducer) is expressed in endothelial cells and serves as an important modulator in regulating the subcellular localization and activity of endothelial NO synthase [8]. In studies with knock-out mouse models, the loss of Nostrin was associated with restricted migration of endothelial tip cells, endothelial dysfunction, reduced NO synthesis, increased blood pressure and diastolic heart failure [9, 10]. Furthermore, in the zebrafish model, Nostrin knockdown altered the function of the glomerular filtration barrier, resulting in the induction of proteinuria and ultrastructural morphological changes in the glomerular capillary loop [11]. These findings from basic research suggest that Nostrin could play an essential role in the pathogenesis of AKI and we have started to analyze the consequences of loss of Nostrin in the context of kidney diseases in the mouse model. First yet unpublished data from our group suggested the hypothesis that the expression of Nostrin in the serum of patients with AKI could be changed. Therefore we assessed the potential role of serum Nostrin as AKI biomarker in the current study. Three endpoints were defined: in-hospital death, the need of kidney replacement therapy (KRT), and recovery of kidney function (ROKF).

## Materials and methods

### Study population and design

The study was prospective and observational and was conducted at the Brandenburg University Hospital in Brandenburg an der Havel, Germany. The hospital is part of the Brandenburg Medical School and the study was formally approved by the ethics committee of the Brandenburg Medical School. The study was initially designed for the analysis of circulating IL-33 receptor (sST-2) and was later expanded for the analysis of Nostrin and the inclusion of a control group. For this purpose, the ethics committee approved two amendments (No. of initial proposal and amendments: E-01-20190820). The recruitment of participants was conducted between 01.03.2020 and 30.04.2023 and all participants provided written informed consent. Legal guardians of adult patients provided written informed consent if the medical condition of the participant did not allow the signature. In general, the study cohort was the same as reported in a recently published study of our group (Soluble IL-33 receptor predicts survival in acute kidney injury [12]). Therefore, the methods section and the patients´ characteristics are almost comparable between the former and the current investigation. For the control group, adult volunteers were included who had no serious pre-existing medical conditions and no symptoms of disease at the timepoint of blood collection. Other recruited subjects were hospitalized patients treated in different medical departments and were included if the diagnosis AKI was made. It was made if either criterion 1 or 2 of the 2012 published ´KDIGO clinical practice guidelines for acute kidney injury´ [6] was fulfilled. Criterion 3 which describes a reduction in urine output was not considered because information about urine production was missing in too many individuals. Serum Nostrin, NGAL and KIM-1 were measured at the time of AKI onset or diagnosis. All included subjects were at least 18 years old. Pregnancy and the need of a kidney replacement therapy at the timepoint of initial AKI diagnosis were defined as exclusion criteria. Also excluded were patients with pre-existing chronic kidney disease stage 5D according to KDIGO [4], patients with advanced non-malignant or malignant disease and a palliative treatment strategy, and patients with suspected or active COVID-19 disease. The etiology of AKI was either classified according to well-established criteria (sepsis–[13], cardiorenal syndrome types 1 or 3 [14], hepatorenal syndrome [15]) or clinically (obstruction—ultrasound analysis, drug-induced and contrast-associated and post-surgery AKI–patient history). The diagnosis of pre-renal AKI was made in the absence of other causes if patients presented clinical symptoms of dehydration (e.g. dry skin in conjunction with low blood pressure and tachycardia). The criterion ´ventilatory support´ was fulfilled if patients either required non-invasive or invasive ventilation. Exclusive oxygen supply without any type of pressure support was not judged as ventilatory support. DP accessed patient details and clinical outcomes last on 30.06.2023. All other investigators did not have access to clinical information that could identify individual participants. The recommendations of Strengthening the Reporting of Observational Studies in Epidemiology were used [16] (S1 File).

### Blood sampling

The diagnosis of AKI was facilitated by an automated AKI alert system at the Brandenburg University Hospital. Any increase in serum creatinine according to the KDIGO criteria 1 or 2 initiated an automated message to the nephrologist in charge. It contained patient-related information in an anonymized manner. The increase in creatinine, which resulted in the diagnosis of AKI, was reported by the system whenever an increase reached >26.5 μmol/L compared to any previous value within the last 48 hours during the course of hospital therapy. Additionally, the system triggered an AKI alarm if there was a relative increase in creatinine of at least 1.5-fold compared to any previous value within the last 7 days. The baseline creatinine level did not have to be physiological. Every patient provided twice 3.5 mL of blood (serum tubes BD Vacutainer^®^ SST™ II Advance or Sarstedt S-Monovette^®^ serum gel). Blood was drawn in the supine position, venous congestion was minimized. In patients with central venous line blood was collected from there. Blood tubes were stored upright for 30 minutes to maintain the clotting time specified by the manufacturer. This was followed by centrifugation for 10 minutes according to the manufacturer’s instructions. Samples were stored in plastic tubes at constant -20°C until analysis. Blood samples from healthy volunteers in the control group were collected and processed under the same conditions as the patient samples.

### Quantification of serum Nostrin, NGAL and KIM-1

The determination of the concentrations of all investigated parameters was performed from serum samples by sandwich-ELISA technique. The following commercial ELISA kits were used for the analysis: Nostrin (MyBioSource, MBS7608085); Lipocalin-2/NGAL (BioVendor, RD191102200R) and KIM-1 (R&D Systems, DSKM100). All samples were measured in technical duplicates with adjusted dilutions according to the manufacturer’s protocols. Absorbances were determined using a microplate reader (TriStar^®^ S LB 942, Berthold Technologies). Sample concentrations were calculated by generation of the corresponding standard curves and using Four Parameter logistic (4PL) curve fitting (MyAssays online data analysis tool, https://www.myassays.com). Laboratory investigators were blinded to the sample sources and clinical outcomes during the procedures of measurements. Sensitivity and assay range data were: Nostrin—sensitivity 0.188 ng/mL, assay range 0.313–20 ng/mL; NGAL—sensitivity 0.02 ng/mL, assay range 0.03–10 ng/mL, KIM-1—sensitivity 3.63 pg/mL, assay range 10.9–700 pg/mL.

### Endpoints

The following endpoints were defined: in-hospital death, the need of kidney replacement therapy (KRT), and recovery of kidney function (ROKF) until discharge. The second criterion (need of KRT) was fulfilled if at least one KRT session was performed. The following KRT procedures were applied, the respective decisions were made by the nephrologist in charge: hemodialysis (HD), hemodiafiltration (HDF), slow extended daily dialysis (SLEDD), continuous veno-venous hemodiafiltration (CVVHD(F)). ROKF was defined as published by Fiorentino et al. [17]. It was diagnosed if the last serum creatinine concentration did not differ from the initial value by more than 50%.

### Statistics

The results of serum biomarker analysis were tested for normality with the Kolmogorov-Smirnov test. Since data were not normally distributed, the Mann-Whitney test was used for comparisons between two groups. Comparisons between 3 or more groups were performed with the Kruskal-Wallis test. The results are given as mean +/- SD. A p-value of ≤0.05 was defined as statistically significant. Missing values were not inferred. For cut off identification the Youden-Index was calculated based on the ROC curve, which was generated using the variables serum Nostrin (ng/mL) and survival, need of KRT or ROFK. The highest absolute index was chosen as cut off. Statistical analysis were performed with the following applications: WIZARD^®^ for MacOs (Version: 2.0.13, developer: Evan Miller, 2021) and GraphPad Prism^®^ (Version 9.5.0).

## Results

### Baseline characteristics and outcomes

**Fig 1:**
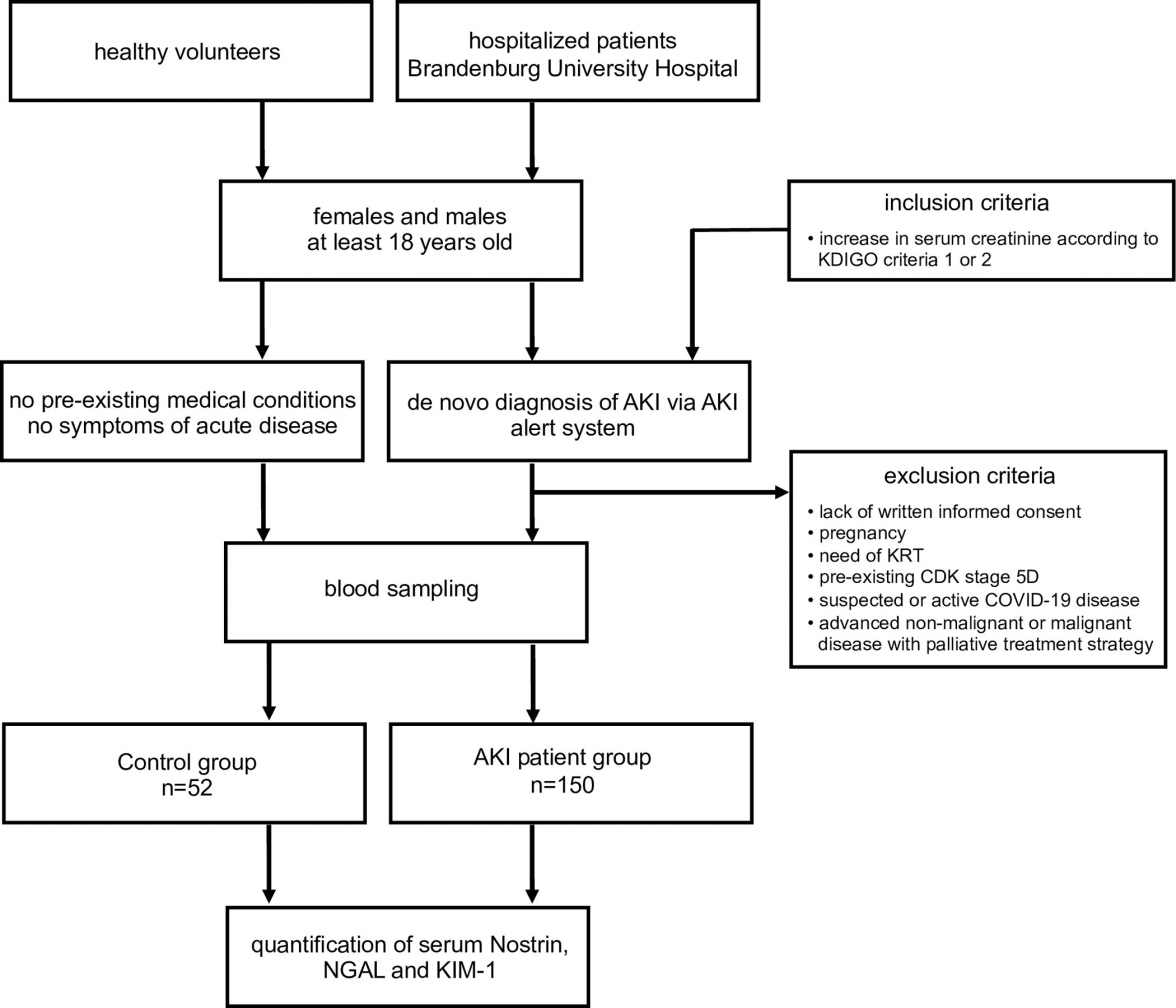
Selection of patients and controls and sample flow.

In total, 150 patients were included in the study (females 62, males 88), the mean age was 75.1 +/- 13.2 years. The in-hospital treatment time was 16.1 +/- 10.8 days. During in-hospital therapy 16.7% of all individuals died, 39.3% required at least one individual KRT session and ROKF was diagnosed in 72%. These and additional patients characteristics are listed in Table 1, which was previously published by our group in a study analyzing soluble IL-33 receptor in acute kidney injury (see also [12]). In addition a total of 52 healthy volunteers were included in the study (females 31, males 21), the mean age was 40.8 +/- 12.4 years. The criteria for selection of patients and controls are illustrated in Fig 1.

**Table 1:** Patients’ characteristics.

variable	result
age (years +/SD)	75.1 +/-13.2
gender (females / males)	62 / 88
in-hospital treatment (days +/-SD)	16.1 +/-10.8
AKI etiology (%)	
sepsis	23.8
volume depletion	23.1
cardiorenal	20.3
contrast-associated	12.6
hepatorenal	2.8
drug-induced	1.4
post-surgery	1.4
obstruction	0.7
combined	13.9
Morbidities	
pre-existing CKD (%)	73.3
arterial hypertension (%)	88.4
diabetes mellitus (%)	47.7
coronary artery disease (%)	42.9
pre-existing heart insufficiency (%)	55.9
pulmonary disease (%)	24.8
obesity (%)	49.3
history of neoplasia (%)	28
KRT initiated (%)	39.3
in-hospital death (%)	16.7
no recovery of kidney function (%)	28
ICU treatment (%)	32
ventilatory therapy (%)	16.7
vasopressor therapy (%)	16.7

### Serum Nostrin, NGAL and KIM-1 in AKI

**Fig 2:**
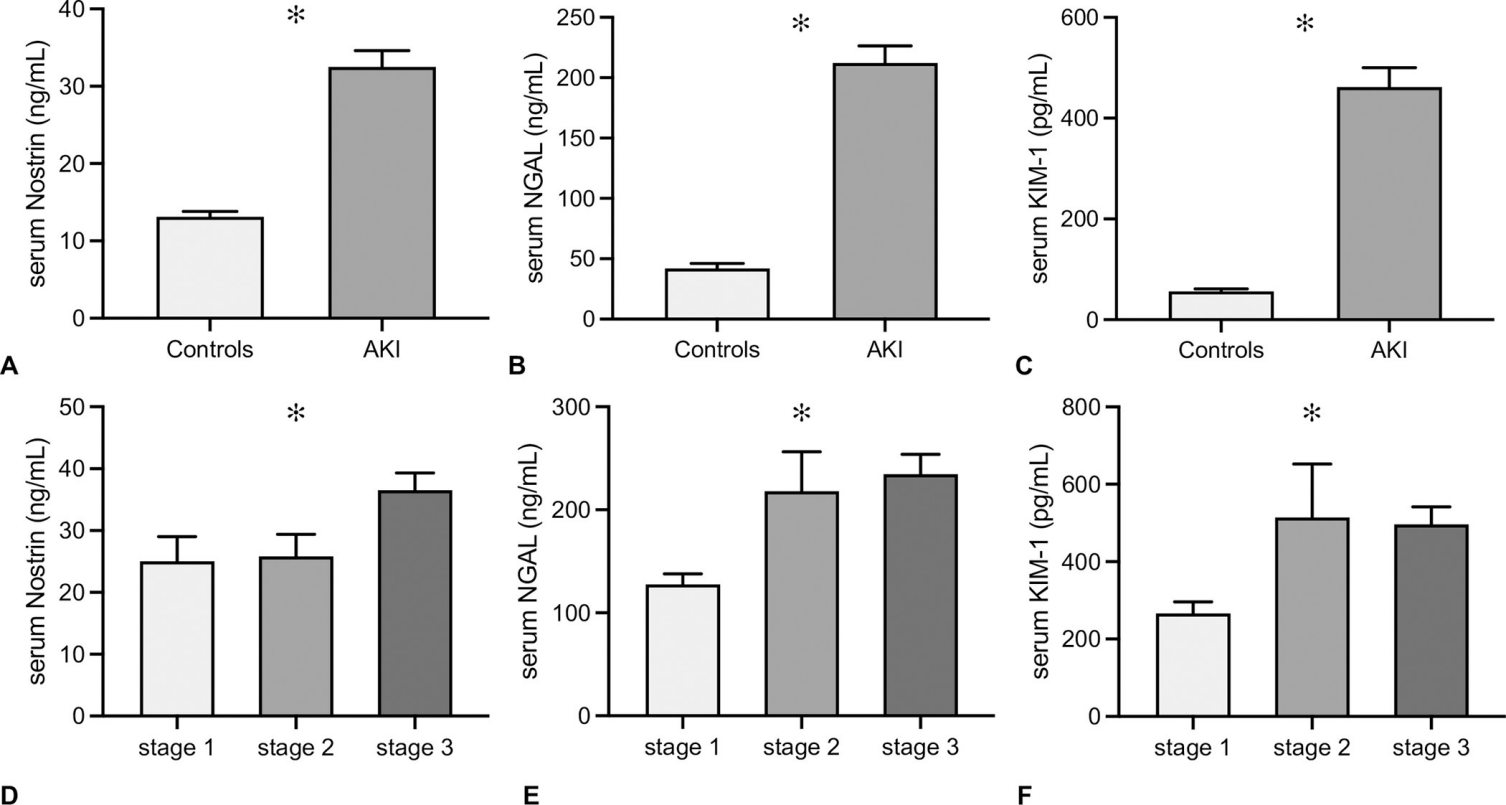
Serum Nostrin concentrations in AKI patients. Serum concentrations of Nostrin (A and D) are shown in comparison to serum NGAL (B and E) and KIM-1 (C and F) in Controls versus AKI subjects (A-C) and in AKI stages 1–3 according to KDIGO 2012 (D-F). All biomarkers were significantly higher in AKI patients than in Controls and Nostrin and NGAL increased with increasing AKI severity according to KDIGO (data in mean +/-SD, *: p≤0.05).

In healthy controls (n = 52), the mean serum concentration of Nostrin was 13.1 +/-5.3 ng/mL, in AKI individuals, it was 32.5 +/-25.7 ng/mL (p<0.001) (Fig 2A). Nostrin serum concentrations gradually increased from AKI stage 1 over 2 to 3 (25.0 +/-4.0 ng/mL vs. 25.8 +/-3.6 ng/mL vs. 36.5 +/-2.8 ng/mL, p = 0.037) (Fig 2D). Both NGAL and KIM-1 significantly differed between controls and AKI patients also (NGAL 33.2 +/-50.6 vs. 212.0 +/-14.2 ng/mL, p<0.001 and KIM-1 46.8 +/-56.3 vs. 461.9 +/-38.1 pg/mL, p<0.001), and AKI stage-related increase was observed for both proteins as well (NGAL stage 1–127.7 +/-10.2 ng/mL, stage 2–218.2 +/-38.1 ng/mL, stage 3 234.6 +/-19.3 ng/mL, p = 0.01, KIM-1 stage 1–205.4 +/-327.3 pg/mL, stage 2–514.3 +/-137.9 pg/mL, stage 3–496.4 +/-45.3 pg/mL, p = 0.036) (Fig 2B, 2C, 2E and 2F). In an additional series of analysis, serum Nostrin / NGAL / KIM-1 were correlated with the estimated glomerular filtration rate (eGFR) according to CKD-EPI [18]. None of the biomarkers correlated with admission or minimal eGFR (Nostrin and admission eGFR p = 0.52; Nostrin and minimal eGFR p = 0.32; NGAL and admission eGFR p = 0.15; NGAL and minimal eGFR p = 0.78; KIM-1 and admission eGFR p = 0.78; KIM-1 and minimal eGFR p = 0.97).

### Endpoints

**Fig 3:**
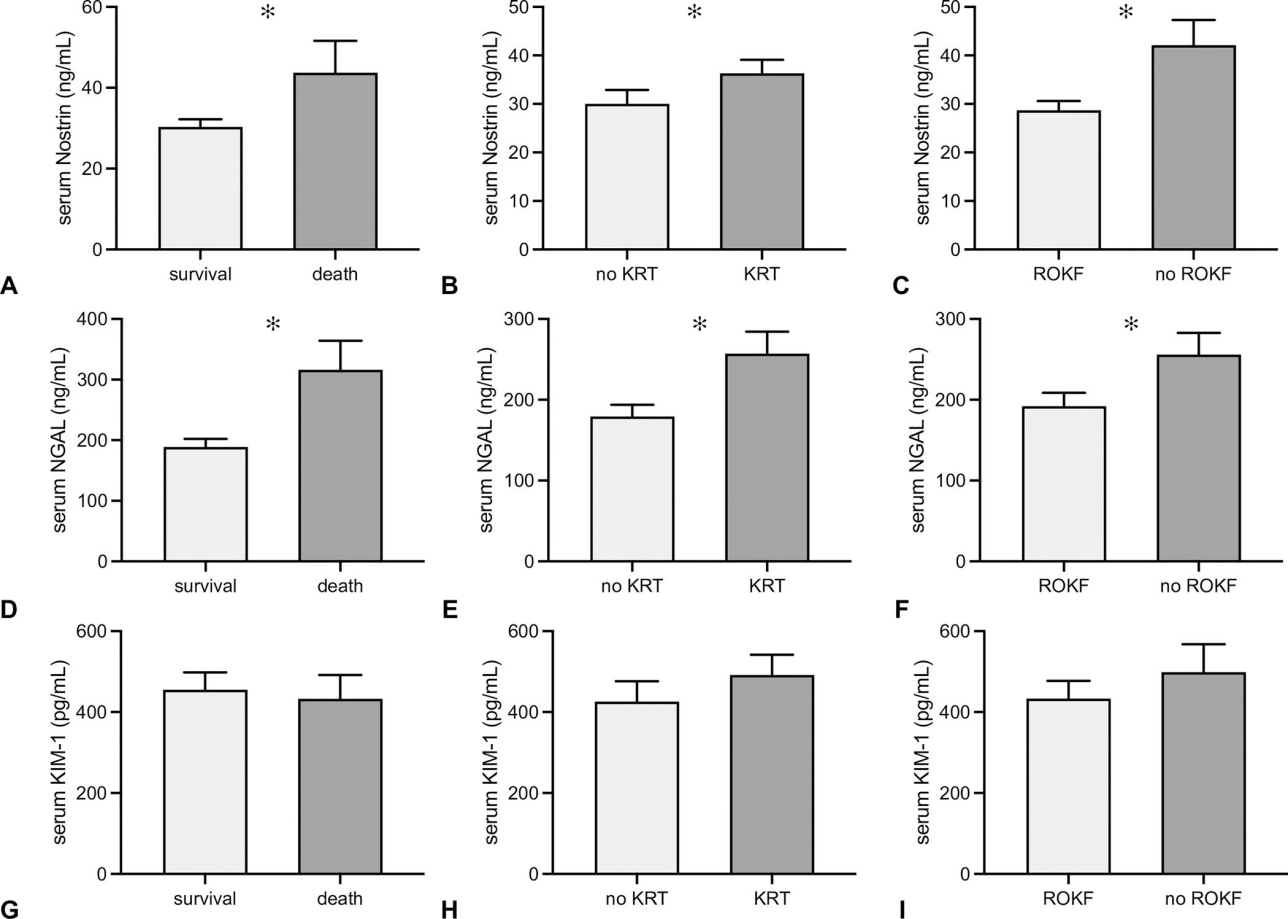
Comparison of serum Nostrin, NGAL and KIM-1 in relation to death, need of kidney replacement therapy and recovery of kidney function. Serum levels of Nostrin (A-C), NGAL (D-F), and KIM-1 (G-I) were evaluated in relation to all primary endpoints (survival, KRT, ROKF). Serum Nostrin, measured at the time of AKI diagnosis was significantly higher in subjects that fulfilled the criteria of the three endpoints. Comparable observations were made for NGAL. KIM-1 however did not differ in any of the endpoint categories (data in mean +/-SD, *: p≤0.05).

Serum Nostrin was lower in survivors than in non-survivors (30.3 +/-1.9 ng/mL vs. 43.7 +/-7.9 ng/mL, p = 0.05, cut off 45.66 ng/mL, sensitivity 40%, specificity 83.2%) (Fig 3A). Patients with the need of KRT during follow-up showed significantly higher serum Nostrin at the time of AKI diagnosis (36.3 +/-2.9 ng/mL vs. 30 +/-2.8 ng/mL, p = 0.004, cut off 17.97 ng/mL, sensitivity 88.14%, specificity 37.36%) (Fig 3B). In addition, individuals without ROKF until discharge showed higher serum Nostrin at the time of AKI diagnosis (42.1 +/-5.2 ng/mL vs. 28.7 +/-1.9 ng/mL, p = 0.009, cut off 45.66 ng/mL, sensitivity 42.86%, specificity 87.96%) (Fig 3C). NGAL analysis showed the same associations, with regard to all three endpoints (death vs. survival 316.1 +/-48.0 vs. 188.8 +/-13.3 ng/mL, p<0.001; need of KRT vs. no need of KRT 257.2 +/-27.2 vs. 179.4 +/-14.5 ng/mL, p = 0.007; no ROKF vs. ROKF 255.8 +/-27.1 vs. 192.2 +/-16.4 ng/mL, p = 0.04) (Fig 3D–3F). KIM-1 on the other hand did not show any associations at all with one of the three endpoints death, KRT, or ROKF (Fig 3G–3I). To evaluate the prognostic values for serum Nostrin, receiver operating characteristic (ROC) curves were generated and the area under the curves (AUC) were calculated and compared to AUC of NGAL and KIM-1. The performance of Nostrin was similar to NGAL whereas the performance of KIM-1 was inferior in comparison to Nostrin and NGAL (Table 2).

**Table 2:** Predictive values of serum Nostrin, NGAL and KIM-1. Data are presented as AUC-ROC values and 95% confidence interval (CI).

outcome	AUC-ROC (95% CI)	AUC-ROC (95% CI)	AUC-ROC (95% CI)
	Nostrin	NGAL	KIM-1
in-hospital death	0.624 (0.450–0.749)	0.668 (0.546–0.790)	0.534 (0.410–0.660)
need of KRT	0.639 (0.551–0.727)	0.615 (0.519–0.711)	0.567 (0.472–0.663)
ROFK	0.637 (0.533–0.741)	0.649 (0.552–0.745)	0.549 (0.442–0.656)

### Etiology

**Fig 4:**
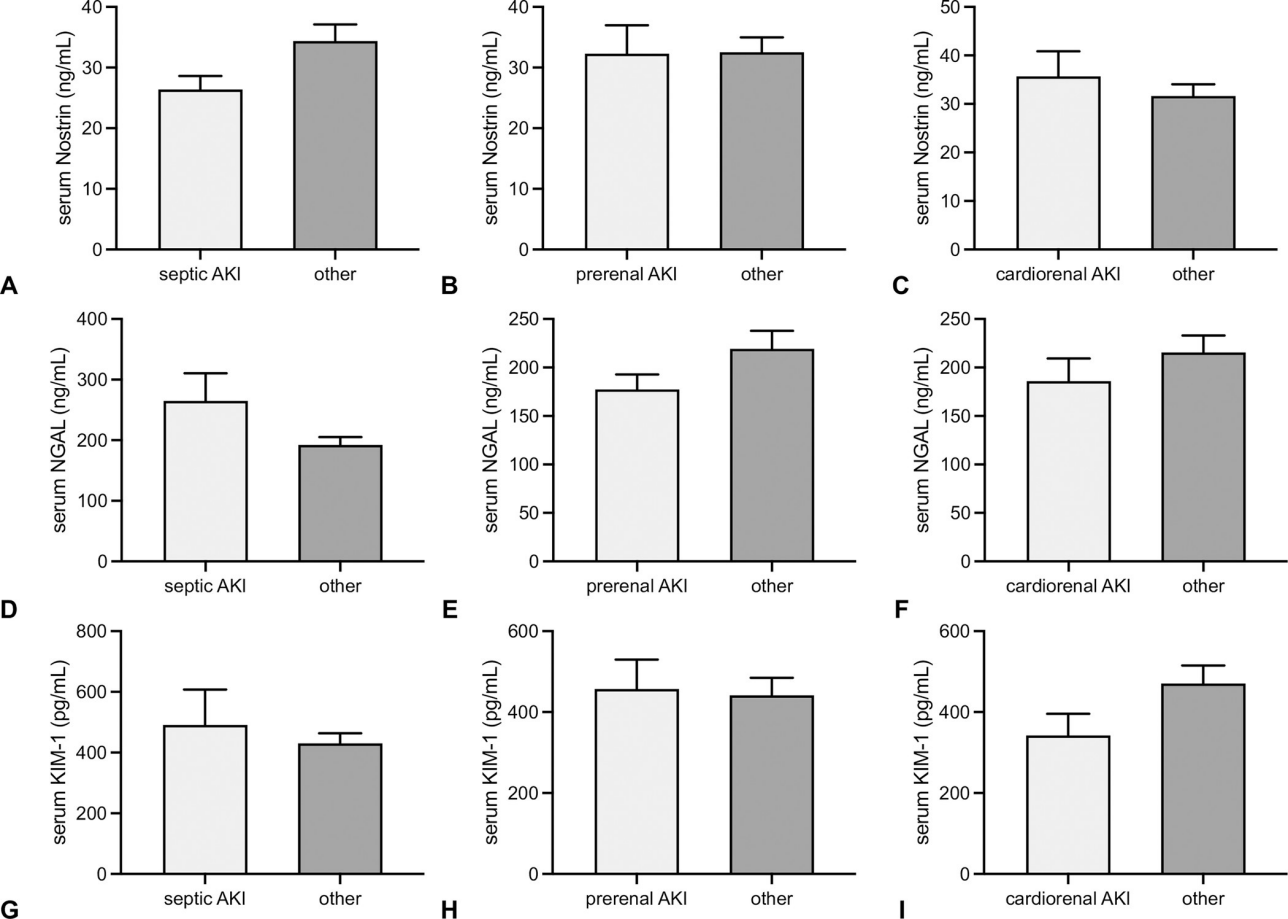
Comparison of serum Nostrin, NGAL and KIM-1 in relation to AKI entities. Differences in serum Nostrin (A-C), NGAL (D-F), and KIM-1 (G-I) in one out of three predefined AKI entities (septic AKI, prerenal AKI, cardiorenal AKI) in comparison to all other types of AKI are shown, respectively. None of the three biomarkers differed in any of the analysis (data in mean +/-SD).

Regarding the AKI cause, serum Nostrin concentrations were not different between any of the entities (p = 0.57). Comparisons between three selected AKI causes with all other AKI types in combination did also not show different serum Nostrin concentrations (septic AKI vs. all other AKI types– 26.3 +/-2.2 ng/mL vs. 34.3 +/-2.7 ng/mL, p = 0.34; prerenal AKI vs. all other AKI types– 32.3 +/-4.6 ng/mL vs. 32.5 +/-2.4 ng/mL, p = 0.48; cardiorenal AKI vs. all other AKI types– 35.6 +/-5.1 ng/mL vs. 31.6 +/-2.4 ng/mL, p = 0.56) (Fig 4A–4C). Serum NGAL and KIM-1 did also not differ between the AKI entities included (Fig 4D–4I).

### Morbidities

Serum concentrations of Nostrin, NGAL and KIM-1 were compared between patients with versus without any of the following pre-existing morbidities: arterial hypertension, coronary artery disease (CAD), diabetes mellitus, heart insufficiency, history of neoplasia, chronic kidney disease, pulmonary disease or obesity. Patients with established CAD and those with a history of neoplasia showed higher serum Nostrin at AKI onset (36.0 +/-2.9 ng/mL vs. 29.8 +/-2.9 ng/mL, p = 0.014 and 41.8 +/-5.6 ng/mL vs. 28.9 +/-1.8 ng/mL, p = 0.018). Serum KIM-1 was higher in subjects without arterial hypertension as opposed to those with hypertension (690.1 +/-168.4 vs. 426 +/-36.1 pg/mL, p = 0.025). Other morbidity-associated differences were not identified for any of the three biomarkers (S1 Table).

### Intensive care therapy

**Fig 5:**
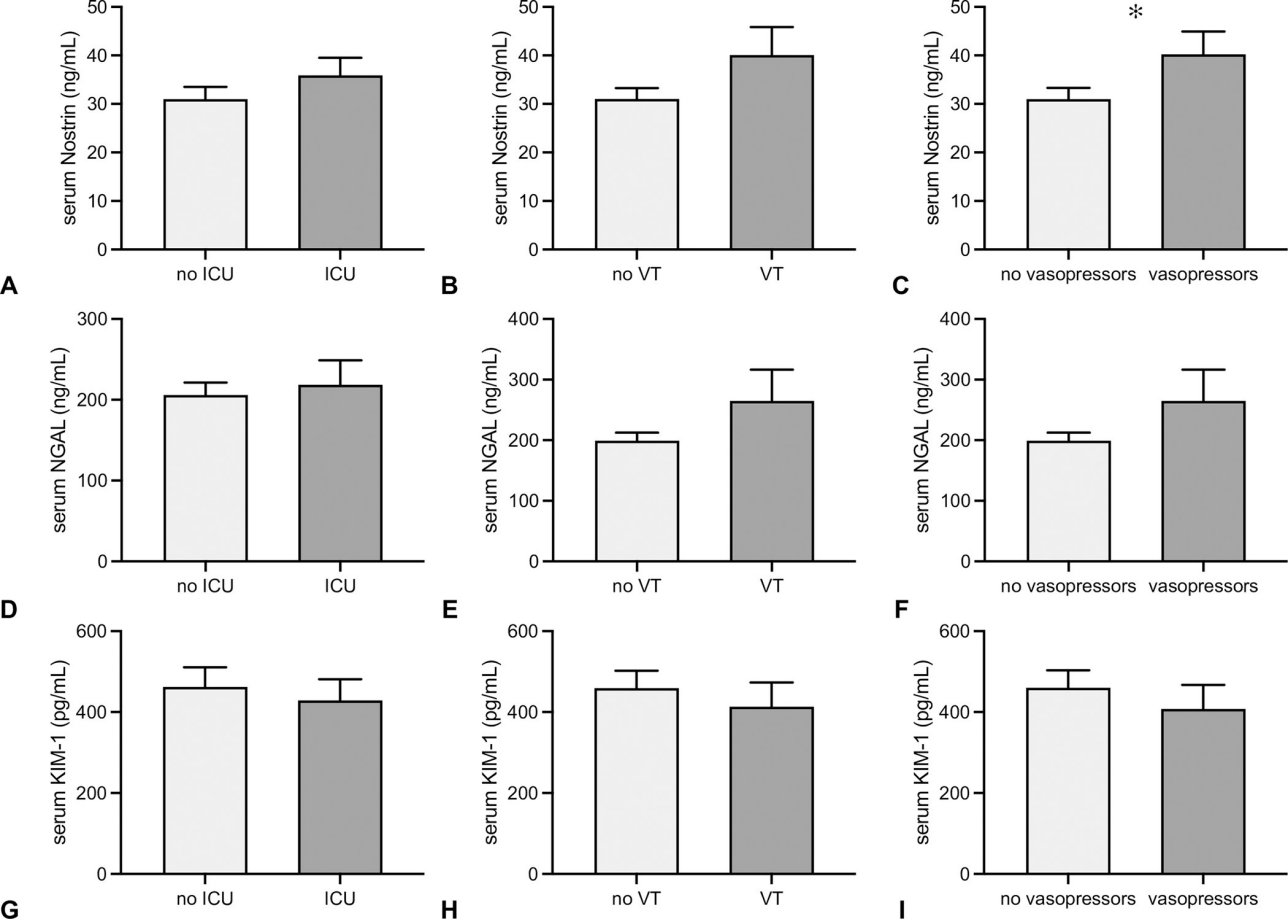
Comparison of serum Nostrin, NGAL and KIM-1 in relation to in-hospital treatment course. Serum Nostrin (A-C), NGAL (D-F), and KIM-1 (G-I) are shown in relation to the need of intensive care therapy, need of ventilatory support and use of vasopressors. Individuals that required ICU therapy in general (A) or with the need of ventilatory therapy (VT) (B) did not show different serum Nostrin as compared to those who did not. The use of vasopressors was associated with higher serum Nostrin (C). Neither NGAL (D-F) nor KIM-1 (G-I) differed in any of the in-hospital treatment categories (data in mean +/-SD, *: p≤0.05).

Three categories were analyzed: intensive care therapy in general, need of ventilatory support and use of vasopressors. Patients treated at the intensive care unit (ICU) did not show different serum Nostrin concentrations than those without ICU therapy (35.8 +/-3.6 ng/mL vs. 30.9 +/-2.5 ng/mL, p = 0.08) (Fig 5A). Also, the need of ventilatory support was not associated with different serum Nostrin levels (40.0 +/-5.7 ng/mL vs. 31.0 +/-2.2 ng/mL, p = 0.08) (Fig 5B). However, subjects that required vasopressor therapy during follow-up showed higher serum Nostrin at the time of AKI diagnosis (40.2 +/-4.6 ng/mL vs. 30.9 +/-2.3 ng/mL, p = 0.007) (Fig 5C). If vasopressor therapy was needed Noradrenaline was used as the first-line vasopressor, possibly supplemented with dobutamine and in rare cases supplemented with adrenaline. Vasopressin has been used in cases of refractory shock. Neither NGAL nor KIM-1 levels were associated with any of the variables intensive care therapy, ventilatory support or vasopressor administration (Fig 5D–5I).

## Discussion

Serum Nostrin measured at the time of AKI diagnosis was shown to be associated with three clinically important outcome variables: in-hospital death, the need of kidney replacement therapy and recovery of kidney function. In addition, the levels of serum Nostrin were generally higher in AKI patients as compared to controls without impaired excretory kidney function. Furthermore, in AKI patients Nostrin serum levels gradually increased in parallel to the AKI stage according to KDIGO [6] and individuals that required vasopressors showed higher Nostrin levels at the time of AKI diagnosis. In previous work we have shown that loss of Nostrin leads to increased blood pressure in the mouse model [10]. Our current hypothesis is that higher Nostrin levels in the serum of patients receiving vasopressor therapy reflect that this group includes possibly individuals with lower blood pressure in general, making these patients potentially more susceptible to a vasopressor therapy in case of AKI. This hypothesis needs to be verified in future studies about the so far unknown molecular mechanisms concerning the increased release of Nostrin to the serum of patients in conditions of acute kidney injury. Regarding all three endpoints only NGAL was predictive comparably to Nostrin. KIM-1 however was not associated with either in-hospital death, or need of KRT, or ROKF. Particularly the KIM-1 related findings are intriguing, since more than 2,000 references on KIM-1 have been published meanwhile (May 2023). As a damage biomarker [7] KIM-1 has significant value in AKI prediction and diagnosis [19, 20]. From the nephrologist´s perspective, the prediction of mortality, KRT and of ROKF is of indispensable value. The study cohort analyzed in this article was the same as published in 2022 [12]. The other analysis, dedicated to soluble IL-33 receptor (sST-2), showed associations between higher sST-2 at AKI onset and mortality, ICU therapy, ventilatory support and the use of vasopressors, respectively. The marker was not predictive with regard to KRT or ROKF. In general, the data on biomarker-based mortality prediction in AKI are limited. We recently reviewed the literature [21] and identified six references that addressed the prediction of in-hospital death in acute kidney injury. Three studies included well known markers such as NGAL, KIM-1, L-FABP, and urinary (TIMP-2) x (IGFBP7) [22–24]. Two studies however evaluated less established biomarkers or marker panels (serum fibrinogen level to the albumin level ratio (FAR) [25] and metabolic profiles [26]). A ROKF predictive role has been shown for serum proenkephalin A [27], urine C–C motif chemokine ligand 14 [28], and for several targets of proteomic studies [29]. Recovery prediction is directly linked to the identification of Acute Kidney Disease (AKD), which must be diagnosed if kidney excretory dysfunction persists for longer than 7 days [5]. AKD potentially reflects the transition from AKI to CKD. Chawla and colleagues [5] reported on ´Acute kidney disease and renal recovery´ in 2017. They stated that ´Early identification of persistent AKI is important in order to initiate an extended evaluation and management protocol to avoid further kidney damage and associated mortality.´ Subsequently, a 2017 published study by Kellum et al. [30] was mentioned, which included almost 17,000 AKI patients who were analyzed for post-AKI recovery status in relation to outcomes after discharge. At year 1 after discharge, mortality was significantly higher in subjects who did not recover completely as opposed to those with so-called early reversal. The mortality risk after discharge also depends on AKI severity as discussed by Rewa and colleagues [2]. Thus, the earliest possible recognition of individuals at risk of incomplete or no recovery at all is of the highest importance with regard to mortality reduction and it has been emphasized, that AKI patients with an urgent need of KRT in the nearest future should be identified earlier rather than receive KRT earlier than necessary. Serum Nostrin can potentially help to close the diagnostic ´gap´ or to reduce the uncertainty on whether patients will require KRT or not. An inherent limitation, albeit challenging to avoid, is the definition of AKI used in the study. Based on KDIGO criteria, which rely on dynamic changes in creatinine levels, the late responsiveness of creatinine as a biomarker limits early AKI detection. A further limitation of the study is that we were not able to consider urine volumes since respective information was missing in too many individuals. Furthermore, we measured serum Nostrin concentrations at the timepoint of initial AKI diagnosis only and the study lacks long-term follow-up data. Therefore we are not able correlate serum Nostrin levels with patients with oliguria or anuria. Typically, studies on AKI in the intensive care setting include survival data at day 60 or 90, providing insights into renal recovery over longer periods post-diagnosis. If renal dysfunction persists for more than 7 days AKI transitions to AKD, putting individuals at higher risk of developing CKD. Having data up to 3 months could accurately assess the transformation rate to CKD. Nostrin might have predictive value in this regard and further studies with long-term evaluation and larger group sizes are planned for our future research. A key strength of the study is the comparison with NGAL and KIM-1. Nostrin even demonstrated a certain superiority over KIM-1. However, the true strength lies undoubtedly in the identification of a new, highly promising predictor of clinically relevant endpoints of AKI. An association of Nostrin with the etiology of AKI was previously unknown. The present study provides a promising basis for further investigations into the mechanistic understanding of the role of Nostrin in AKI and could provide new starting points for therapy and prevention. If it was to be decided whether Nostrin either represents a marker of impaired function or damage, the second category appears more likely. Firstly, we did not find any correlation with the estimated GFR. Secondly, the protein is significantly expressed by endothelial cells. In AKI three pathophysiological events take place on an almost regular basis: tubulopathy, interstitial inflammation [31] and microvasculopathy [32]. Microvasculopathy typically affects the peritubular and glomerular capillary network and several processes are involved including endothelial leukocyte adhesion, microvascular thrombosis, endothelial inflammation and hypoxia-induced endothelial cell swelling [33, 34]. In addition, the microvasculature suffers from permanent structural damage, ultimately reflected by the loss of total capillary diameter [35, 36]. The latter has been associated with an increased risk of chronic kidney disease in the long-term [36, 37]. Regarding the robust endothelial expression of Nostrin in conjunction with its role in maintaining the NO metabolism, we currently suppose Nostrin as AKI damage biomarker. It needs however to be elucidated whether the protein also suits as diagnostic parameter beside the role in AKI risk prediction.

In summary, serum Nostrin was identified as new predictor of three important clinical outcome variables in acute kidney injury. Future investigations will focus on long-term evaluation, expansion of the sample size and will also address the question on whether the protein is suitable for AKI prediction per se (e.g. in sepsis/septic shock or heart failure).

## Supporting information

S1 File Completed STROBE checklist for cohort studies. (PDF)

S1 Table Raw data of biomarker measurements. The table lists all values of serum Nostrin, NGAL and KIM-1 measurements and corresponding characteristics of AKI patients and controls.
(XLSX)
