# Supplementary material for: Serum Nostrin—A risk factor of death, kidney replacement therapy and acute kidney disease in acute kidney injury
Source: PLoS One. 2024 Apr 11;19(4):e0299131. doi: 10.1371/journal.pone.0299131 (PMC11008819; doi:10.1371/journal.pone.0299131)
Supplement: S1 File — (PDF) [file pone.0299131.s001.pdf]

S1 Table. Completed STROBE checklist for cohort studies.

	Item No	Recommendation	Respected ?	Comments
**Title and abstract**	1	(*a*) Indicate the study's design with a commonly used term in the title or the abstract	Yes	The study design is indicated in the "Design, setting, participants, & measurements" section of the abstract.
		(*b*) Provide in the abstract an informative and balanced summary of what was done and what was found	Yes	These information are provided in the "Design, setting, participants, & measurements" and "Results" sections of the abstract.
**Introduction**				
Background/rationale	2	Explain the scientific background and rationale for the investigation being reported	Yes	The scientific background and the literature about the rationale to measure serum Nostrin as novel biomarker in AKI patients are summarized in the introduction section.
Objectives	3	State specific objectives, including any prespecified hypotheses	Yes	The last sentences of the introduction specify as goal of the study to assess the potential role of serum Nostrin as AKI biomarker in regard of the endpoints: in-hospital death, the need of kidney replacement therapy (KRT), and recovery of kidney function (ROKF).
**Methods**				
Study design	4	Present key elements of study design early in the paper	Yes	The study population and design is the first section of the Material and methods. The first sentence of Material and methods describes that the study was prospective and observational.
Setting	5	Describe the setting, locations, and relevant dates, including periods of recruitment, exposure, follow-up, and data collection	Yes	The setting and the location of the study at Brandenburg University Hospital as well as the periods of recruitment of participants and the access of patient details and clinical outcomes are described in the study population and design section of the Material and methods.
Participants	6	(*a*) Give the eligibility criteria, and the sources and methods of selection of participants	Yes	The study population is described the "study population and design" section of the Material and methods and also the inclusion and exclusion criteria are described. Furthermore, Figure 1

				summarizes the selection of patients and controls and the sample flow of the study.
Variables	7	Clearly define all outcomes, exposures, predictors, potential confounders, and effect modifiers. Give diagnostic criteria, if applicable	Yes	The outcomes of the study are defined in the "Endpoints" section of the Material and methods as: in-hospital death, the need of kidney replacement therapy (KRT), and recovery of kidney function (ROKF) until discharge. Diagnostic criteria for KRT and ROFK are described in the same section.
Data sources/ measurement	8*	For each variable of interest, give sources of data and details of methods of assessment (measurement). Describe comparability of assessment methods if there is more than one group	Yes	The section "Blood sampling" of the Material and methods describes how the blood samples of the AKI patients and Controls were collected and processed. The section "Quantification of serum Nostrin, NGAL and KIM-1" describes the technical procedures of ELISA measurements and calculation of the serum concentrations of all three biomarkers analysed within the study.
Bias	9	Describe any efforts to address potential sources of bias	Yes	We tried to reduce bias by blinding laboratory investigators to the sample sources and clinical outcomes during the procedures of measurements. This is stated in the section "Quantification of serum Nostrin, NGAL and KIM-1" of the Material and methods.
Study size	10	Explain how the study size was arrived at	Yes	The period of recruitment of participants as well as the inclusion criteria of participants is described in the section "Study population and design" of the Material and methods.
Quantitative variables	11	Explain how quantitative variables were handled in the analyses. If applicable, describe which groupings were chosen and why	Yes	The section "Quantification of serum Nostrin, NGAL and KIM-1" of the Material and methods describes that all samples were measured in technical duplicates and that concentrations were calculated by generation of the corresponding standard curves and using Four Parameter logistic (4PL) curve fitting.
Statistical methods	12	(*a*) Describe all statistical methods, including those used to control for confounding	Yes	The section "Statistics" of the Material and methods describes all applied statistical tests and software tools used.
		(*b*) Describe any methods used to examine subgroups and interactions	N/A	Non applicable
		(*c*) Explain how missing data were addressed	Yes	In the section "Statistics" of the Material and methods section is stated that missing values were not inferred.

		(*d*) If applicable, explain how loss of follow-up was addressed	N/A	Non applicable
		(*e*) Describe any sensitivity analyses	N/A	Non applicable
**Results**				
Participants	13*	(a) Report numbers of individuals at each stage of study—eg numbers potentially eligible, examined for eligibility, confirmed eligible, included in the study, completing follow-up, and analysed	Partly	The total numbers of finally included and analysed AKI patients and Controls are provided in the results section and in Figure 1. Numbers of excluded individuals at different stages were not documented.
		(b) Give reasons for non-participation at each stage	Mostly	The reasons for non-participation are defined as overall reasons of the whole study setting in the exclusion criteria. The exclusion criteria are described the section "study population and design" of the Material and methods.
		(c) Consider use of a flow diagram	Yes	Figure 1 shows a flow diagram of selection of patients and controls.
Descriptive data	14*	(a) Give characteristics of study participants (eg demographic, clinical, social) and information on exposures and potential confounders	Yes	Table 1 describes all patients characteristics.
		(b) Indicate number of participants with missing data for each variable of interest	Mostly	Supporting information table S2 lists all values of biomarker measurements and corresponding characteristics of AKI patients and controls and also provides information about missing data.
Outcome data	15*	Report numbers of outcome events or summary measures	Yes	All numbers are either reported in the text of the results section or listed in Table 1 and Table 2.
Main results	16	(*a*) Give unadjusted estimates and, if applicable, confounder-adjusted estimates and their precision (eg, 95% confidence interval). Make clear which confounders were adjusted for and why they were included	Yes	All main results of the quantitative ELISA measurements of all biomarkers are presented in Figures 2-5 as mean +/-SD and Table 2 reports the predictive values of serum Nostrin, NGAL and KIM-1 in respect to the outcomes in-hospital death, need of KRT and ROFK. Data are presented as AUC-ROC values and 95% confidence intervals are reported.
		(*b*) Report category boundaries when continuous variables were categorized	N/A	Non applicable

		(*c*) If relevant, consider translating estimates of relative risk into absolute risk for a meaningful time period	N/A	Non applicable
Other analyses	17	Report other analyses done—eg analyses of subgroups and interactions, and sensitivity analyses	Yes	The cut off values of serum Nostrin concentrations in regard to in-hospital death, need of KRT and ROFK were calculated and are provided in the text of the section "Endpoints" and the numbers of sensitivity and specificity are reported.
**Discussion**				
Key results	18	Summarise key results with reference to study objectives	Yes	The key results about serum Nostrin as novel biomarker in AKI patients are summarized at the beginning of the discussion section.
Limitations	19	Discuss limitations of the study, taking into account sources of potential bias or imprecision. Discuss both direction and magnitude of any potential bias	Yes	The limitations of the study are listed and judged in the discussion section.
Interpretation	20	Give a cautious overall interpretation of results considering objectives, limitations, multiplicity of analyses, results from similar studies, and other relevant evidence	Yes	The interpretation of the results was carried out in comparison with the current state of research and citing the relevant literature.
Generalisability	21	Discuss the generalisability (external validity) of the study results	Yes	The potential impact of the results of the study for future investigations are mentioned in the discussion section.
**Other information**				
Funding	22	Give the source of funding and the role of the funders for the present study and, if applicable, for the original study on which the present article is based	Yes	The funding information were provided upon submission of the manuscript but not included in the manuscript itself as requested by the guidelines of the journal.

*Give information separately for exposed and unexposed groups.
